# Understanding Ethical Concerns Involving Vulnerable Human Participant Populations in Medical Research: Mixed-Method Analysis of Liberian Ebola Survivors’ Experiences in PREVAIL I–VII

**DOI:** 10.3390/healthcare12191989

**Published:** 2024-10-05

**Authors:** Jessi Hanson-DeFusco, Decontee Davis, Meghana Bommareddy, Zainab Olayemi Olaniyan

**Affiliations:** 1 Humanities, Social Sciences, and Communication Department, College of Arts and Sciences, Lawrence Technological University, Southfield, MI 48075, USA; 2 Winifred J. Harley College of Health Sciences, United Methodist University of Liberia, Monrovia 1000, Liberia; ddecontee54@gmail.com; 3 Public Policy and Political Economics Program, School of Economic, Political, and Policy Sciences, University of Texas at Dallas, Richardson, TX 75080, USA; meghana.bommareddy@utdallas.edu (M.B.); zainab.olaniyan@utdallas.edu (Z.O.O.)

**Keywords:** clinical trials, Ebola survivors, global health, ethical treatment, medical research

## Abstract

**Background:** As the number of large-scale outbreaks continues to rise worldwide, clinical trials are increasingly engaging disease-affected peoples within the Minority World (nations with over 80% poverty rates). Yet global health research inadequately addresses potential ethical issues of including impoverished, disease-affected populations and their contextual vulnerabilities in medical research. **Objective:** This paper presents a mixed-method analysis from our 2022 semi-structured survey capturing the experiences of Liberian Ebola Virus Disease (EVD) survivors serving as study participants in the Partnership for Research on Ebola Virus in Liberia (PREVAIL) clinical trials. **Methods:** Firstly, we conducted an extensive literature review of the scholarship related to biomedical research and ethical standards protecting study participants to inform our survey method and design. Applying a theoretical framework on vulnerability, we then qualitatively explored the survey responses of 19 EVD survivors’ perceptions and experiences taking part in PREVAIL, including their expectations, treatment, delivered benefits, and quality of care. We further quantitatively codified their statements for underlying themes of reported negative experiences against standard ethical regulations in biomedical research, conducting a statistical analysis to inform generalizability. Most of the 19 survivors reported facing extreme ongoing vulnerabilities related to their disease status, including physical impairments, psychosocial stress, and socio-economic inequity. **Results:** Initially, the survivors tended to experience a sense of hope and pride in volunteering for PREVAIL. One in five participants reported benefiting from PREVAIL’s regular medical diagnoses. However, most of their survey responses indicated prevalent negative shared experiences, including continually being confused or misinformed of their study participant rights, roles, and benefits; being burdened by heavy participation transaction costs; and repeated incidents of poor treatment and discrimination by PREVAIL staff after initial recruitment. PREVAIL participant satisfaction ranking is negatively correlated with receiving insufficient financial compensation (r = −0.51), extensive time requirements for each medical visit (−0.40), and being poorly treated by clinical staff (−0.67).

## 1. Introduction

Human participant research plays an ever more vital role in medical breakthroughs to combat contagious diseases [1], especially as the number of global epidemiological threats continues to grow exponentially in recent decades [2]. Large-scale outbreaks can offer researchers access to rare disease-affected populations, including survivors as human participants in clinical trials to aid in medical advancements [1,3,4]. One of the worst pre-COVID-19 health crises was the 2014–2016 West Africa outbreak, the largest recorded Ebola epidemic since 1976 [5,6]. Liberia, one of eleven nations affected, experienced 10,678 Ebola cases with a 55% survival rate [7,8,9]. At the end of the West African crisis, there was an estimated 17,000 West African Ebola Virus Disease (EVD) survivors, with approximately 5000 survivors residing in Liberia [10,11,12].

The global shock of the 2014–2016 West African outbreak quickly led to an exponential increase in international support and funding for Ebola biomedical research, from USD 3 million/year before 2015 to USD 116 million between 2015 and 2016 [13,14,15,16,17]. In October 2014, the Partnership for Research on Vaccines and Infectious Diseases in Liberia (PREVAIL) was launched as a large-scale biomedical research initiative, implemented by multiple stakeholders like the Government of Liberia with sponsorship by the US National Institute of Allergy and Infectious Diseases (NIAID). Starting in February 2014, PREVAIL recruited approximately 1500 Liberian survivors and 6000 of their close contacts as human participants in randomized vaccination trials, physical and bodily fluid exams, psychosocial assessments, and natural studies over the next five years [10,18,19,20]. This study examines the shared experiences of Liberian EVD survivors as human participants in PREVAIL.

### 1.1. EVD Survivor Participation in PREVAIL

As demonstrated by various HIV/AIDS studies, human participant involvement can offer unique opportunities to people negatively impacted by their disease status and related health impairments to altruistically give of themselves to a greater cause, so that their suffering and loss may not be in vain [4]. Starting in 2014, in meetings with the Liberian Ministry of Health (MOH) and the Ebola Survivors’ Network, hundreds of EVD survivors sought opportunities to contribute to any response or research efforts. They were often motivated by the hope of finding a sense of purpose to cope with the effects of survivor’s guilt and a need to feel useful. Others wanted to become part of a team comprised of empathetic survivors like themselves with similar shared experiences [5,21,22,23,24,25,26,27]. Additionally, most volunteer opportunities came with supplemental benefits like financial stipends and potential for future employment [11,23,27].

In turn, the 2014–2016 outbreak offered medical researchers with the unique prospect of accessing EVD survivors on a large scale to robustly study the effects of Ebola [15,17,28]. PREVAIL initiated its first clinical trial in 2015, and as financial support continued to increase, seven staged studies were executed in total, using different methods, target populations, and sample sizes [10,18,19,20]. Throughout its various clinical trials, the PREVAIL design included screening all recruited participants for a history of family illness, conducting a physical exam, and performing blood tests. Some were also provided eye examinations. Ebola survivors and those who entered an ETU but may not have had Ebola then were asked to visit a PREVAIL clinic at 3, 6, and 12 months, followed by 6-month visits over 5 years [10,13,14]. A formal research report on PREVAIL notes its various contributions to survivors’ longer-term recovery. For instance, the PREVAIL VII clinical trial (evaluating the persistence of Ebola viral RNA in the eye and response to cataract surgery among EVD survivors) concluded that “EVD survivors and controls demonstrated significant visual improvement from cataract surgery” [17] (paragraph 3). Likewise, PREVAIL I, II, IV, and V are reported to have led to crucial epidemiological advancements, including the provision of vaccines against the disease [13,14,29] (list of all staged studies and contributions/key findings summarized in Appendix B). However, there are initial reports of poor implementation and procedural oversight quality and criticism by EVD survivors pertaining to PREVAIL [9,15,29,30,31].

### 1.2. Ethical Concerns of Biomedical Research and Vulnerable African Populations

In the past 50 years, “the level of oversight on human participants research has exploded from almost none to what is now an exhaustive system of protections. Still, these oversights and protections are not uniform throughout the world, and many nations, especially poor-resource nations, remain at risk for ethical improprieties” [1] (paragraph 3). Ethical issues in medical research in the Majority World (countries with at least 80% poverty rates) continue to gain attention. Cases include McGown’s testing of new drugs and anesthetics without proper authorization in Zimbabwe in the 1990s to the 1996 Pfizer American testing of trovafloxacin against ceftriaxone to treat bacterial meningitis killing eleven Nigerian children [32,33,34,35,36]. Yet despite “documented reports of research abuses in Africa and poor-resource nations in other parts of the world, the majority of incidences related to breaches in ethical conduct most likely go unreported” [33] (p. 961). Additional factors diminishing a person’s quality of life (QoL) (e.g., extreme poverty, gender inequity, low education) can make them more susceptible to being less informed of their rights and roles; taken advantage of; and/or inadequately protected from harm, especially when the ethical standards and procedures of a study do not account for their contextual needs [36,37,38,39].

Survivors entering PREVAIL tended to face a variety of vulnerabilities. Nearly all Liberian survivors, many of whom came from low-income and low-educational backgrounds, were made more vulnerable by their disease status. Most adult survivors continually struggled with long-term EVD-related physical impairments (e.g., blindness, nerve damage, body pains), poor mental health (e.g., depression, PTSD), and increased socio-economic hardships (e.g., being fired, abandoned, or evicted due to disease status) [23,26,40,41]. PREVAIL further confirmed these vulnerabilities in its third trial, which “determine[d] the long-term consequences of EVD by comparing health outcomes in survivors (966 people) and a control group of uninfected household and community contacts (2350 people)” [42] (p. 1). Yet while there were reports about negative experiences of EVD survivors in medical research, there are few formal investigations linked to their vulnerabilities. We aim to address this research gap by examining Liberian EVD survivors’ reported experiences and perspectives as recruited human participants in PREVAIL.

### 1.3. Rationale and Study Purpose

With this background, our research project applied a mixed method design conducting our 2022 semi-structured survey of 19 Liberians EVD survivor respondents. Applying a theoretical framework of vulnerability, we examined two key questions:

- What are potential underlying themes of EVD survivor experiences as human participants in PREVAIL I–VII?
- To what extent is EVD survivors’ quality of life affected by their experiences with PREVAIL?

Our research team first conducted a robust literature review related to biomedical research, human participant involvement, ethics, and Ebola to inform our study design, method, and research framework. Based on the literature and initial PREVAIL and partner reports (such as by USAID/CDC), we hypothesized the following:

**H1:**  Ebola survivors’ participation in PREVAIL is linked to improved self-efficacy and psychosocial health.

**H2:**  Survivor participants gained targeted medical support for their specific disease status at PREVAIL clinics.

**H3:**  Any negative experiences involving biomedical research ethical standards during PREVAIL negatively impacts human participants’ quality of life.

This article is presented as follows. We first detail our theoretical framework, materials, and methods. We then summarize the qualitative results of common shared experiences reported by 19 EVD survivor respondents of our digital semi-structured survey. Secondly, we supplement the analysis by codifying open-ended statements related to key threads of their experiences against ethical standards for protecting human participants in medical research and then perform a statistical analysis (correlation; bootstrap) to address the generalizability of their experiences against the larger PREVAIL human participant population. We elaborate on the survey results in reference to key findings from our literature review in Section 4 and, lastly, present research implications and recommendations in Section 5.

### 1.4. Theoretical Framework

This research applied the theoretical framework on vulnerability, which offers both theoretical and practical foundations for ethical human subject research as presented by Gordon (2020). Vulnerable participants require extended protections to safeguard their rights and wellbeing. Vulnerability can be theoretically studied by two approaches; the categorical approach examines which groups and populations are vulnerable, and the contextual approach characterizes various vulnerabilities that may affect the individual person. Study participants in medical research can be made vulnerable in a series of different ways, which, when aggregated, can negatively impact their involvement in biomedical interventions [38]. There are six types of vulnerabilities:

- Medical—involving physical impairments, poor mental health, and poor medical services/access
- Economic—inequitable distribution of social goods and services like financial income or secure housing
- Social—when an individual falls into an undervalued social group because of their health status
- Cognitive—diminished capacity or inability to effectively communicate
- Institutional—vulnerability to hierarchical power dynamics
- Deferential—informal hierarchical power dynamics such as those created by gender or social economic status; imbalances in power dynamics like doctor and patient, or knowledge like researcher and low-educated participants [38].

Ethical codes of medical research specifically concern the need for proper safeguards and operational protocols to be put into place to address all study participant and/or medical patient needs related to their vulnerabilities [37,38,43].

## 2. Materials and Methods

### 2.1. Study Design

During January–April 2022, our research team conducted a robust literature review of 331 top-cited scholarship on Google Scholar and PubMed, using key search terms including Ebola, biomedical research, PREVAIL, Liberia, ethical standards, participants in clinical trials, development, and epidemiology to inform the study purpose and design. We additionally mapped standard ethical regulations in biomedical research and medical treatment, applying a theoretical framework on vulnerability in healthcare.

In April, the survey was designed by the research team, including being formally reviewed by a team of Liberian global health experts from the Ministry of Health and University of Liberia to ensure the instrument was contextually relevant and culturally sensitive. The primary investigator (PI) worked as an EVD responder with the MOH, MOG, and ESNL throughout the Liberian crisis. This study builds on some of her previous research on EVD-affected peoples [5,41]. Additionally, one of the co-investigators is an EVD survivor with medical training and field survey experience of EVD survivors.

The survey includes open-ended questions, as well as Likert-scaled ranking questions, capturing both quantitative data and testimonies of their post-EVD-recovery experiences including participating in PREVAIL, governmental support, and sentiments/motivational statements related to their disease status (see Appendix A). The survey used both standard English and Liberian English. A key scale incorporated was the Perceived Psychosocial Stress Scale, which was widely used during the 2014–2016 EVD outbreak [5,27,41]. It captures self-reported stressors like anxiety, withdrawal, anger, and flashbacks [44,45,46]. The survey was pilot tested with alpha testing on Likert scaling (α > 0.65) to verify high inter-rater reliability. All pilot test data were excluded.

The survey was launched on Qualtrics (2023, SAP, USA) through May–July 2022 using a snowball approach on the Liberian Ebola Survivor group and Ebola Information Facebook pages. Snowball sampling (or chain-referral sampling) is a nonprobability sampling method in which initial study participants share the study with acquaintances to expand recruitment. Use of social media in snowballing can be an effective means of facilitating the distribution of the digital survey to hard-to-reach populations [47,48,49]. An estimated three-in-four survivors use Facebook to communicate with other survivors and survivor information pages [29], thus serving as a strong means of recruiting survey participants.

The digital survey included informed consent that was presented first after clicking on the survey, which participants read and then clicked on “agree to participate” to continue with the survey. The nature of the study required participants to have some level of technology to complete the survey. Eligibility criteria included speaking English; basic literacy; being a Liberian citizen; having documentation of being a survivor of EVD; and being aged 18–65. Participation was anonymous.

### 2.2. Data Collection and Analysis

During data collection, 33 people clicked on the survey link, with 25 completing the survey on Qualtrics. Six participants’ data were excluded due to the participants not having consented or being non-survivors. In total, 19 adult survivor testimonials were included (13 women/6 men, ages 25–49, residing in Montserrado and Margibi (urban/semi-urban counties)).

**Figure 1:**
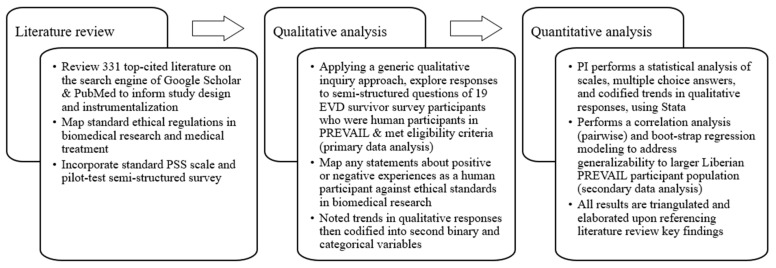
Data analysis phases.

The first phase of the data analysis was a literature review of 331 top-cited sources, which informed the study design, as well as later analysis of the survey data (see Figure 1). In the second phase, the self-reported responses of the survivors who participated in our survey were qualitatively explored by the entire research team, facilitated by the PI. We employed a generic qualitative inquiry approach, commonly used in mixed method psychological studies using a survey design. Unlike a phenomenological approach, generic qualitative inquiry collects data using semi-structured questions capturing qualitative statements in which participants share their ideas and experiences of real events [50,51,52]. Compared to the better-known qualitative approaches like phenomenology, grounded theory, or ethnography, the generic qualitative inquiry approach “is clearest when it is defined in the negative: it is research that “is not guided by an explicit or established set of philosophic assumptions in the form of one of the known [or more established] qualitative methodologies” [53] (paragraph 9). Generic studies epistemologically apply a social constructivist lens that explores how participants interpret their personal lived experiences, conceptualize their world/environment, and give meaning to these experiences [51,54]. Studies using this approach can offer rich descriptions of the phenomenon being studied and are commonly quite inductive and include open codes, categories, and thematic analysis [51,55].

We adopt the process recommended by Percy, Kostere, and Kostere (2015) (pp. 76–84) [50]. Qualitative data (open-ended responses) can then be re-structured quantitatively, often converting them into a second binary or categorical variables, based on noted trends (shared experiences or similar opinions/perspectives) among various participant statements. Like how ethnographers and field researchers code their field notes, this approach can allow for a statistical analysis to support qualitative analysis [50,51,52,56,57,58,59]. The purpose of this additional step is to measure the frequency of trends and to assess findings against the general population, especially in studies with limited sampling (small N) [50,51,52,60].

All participant open-ended responses were assessed for naturally occurring trends that were later quantitatively codified. From this, we created binary variables indicating if a person’s statements about their experiences mentioned the following (1 yes; 0 no): having no medical provision, receiving no financial compensation, feeling/experiencing poor treatment by PREVAIL staff, experiencing long visits lasting around eight hours each trip, or feeling they experienced unequal/discriminant opportunities to participate in specific PREVAIL medical treatments or studies like eye exams. We used statistical findings, namely Point Biserial correlation analysis and boot-strap modeling, to triangulate survivor statements. Finally, we explored the relevance of this research, its limitations, and possible ethical implications for global health policy and research. The PI performed the statistical analysis in Stata (StataCorp LLC/SE 17/College Station, TX, USA) with data interpretation support from the research team. Underlying themes are summarized using both quantitative and qualitative data in the Results section.

### 2.3. Ethical Approval

This 2022 research involved a snowball survey approach of Liberians who are Ebola survivors. This project received ethical approval from the University of Texas at Dallas (IRB#22-528).

## 3. Results

### 3.1. Qualitative Analysis

Our literature review indicates that EVD survivors and EVD-affected peoples face challenges that transcend all categories of vulnerability, including increased financial hardships, social stigmatization, EVD-related disabilities like blindness, and decreased mental health (see Table 1). PREVAIL documentation notes many of these vulnerabilities among their study participants [13,14,15,16,17]. This analysis assesses to what extent steps were taken to accommodate and safeguard against these vulnerabilities during the seven staged studies [29,30].

Along with a robust review of the literature, our research team also qualitatively explored the open-ended responses of the 19 survey participants to better understand their experiences as human participants in the PREVAIL trials. Overall, there were five main trends among their statements identified by our research team (summarized in Table 2). We then codified their qualitative statements to assess the frequencies with which the survivor participants reported experiencing or commenting on these threads.

#### 3.1.1. Meeting Survivor’s Long-Term EVD-Related Needs

One of the major topics that the 19 study participants brought up in their responses was their experiences of receiving specialized treatment (or the hope of it) for their long-term physical impairments resulting from suffering EVD. On the one hand, there is recognition of the medical benefits of being a study participant in PREVAIL. All 19 survey respondents mention how PREVAIL offered free diagnostic benefits to all EVD human participants. The diagnostics were linked to baseline and ongoing monitoring of their physical health but were tailored to their special symptoms and needs. They received detailed diagnosis by PREVAIL medical staff, familiar with EVD and its effects on the human body. In total, 10 out of the 19 survey participants (52%) provided statements indicating satisfaction with the free medical diagnoses they received at PREVAIL clinics. This supports the first hypothesis that survivor participants gained targeted medical support for their specific disease status at PREVAIL clinics.

“When I was in prevail it was fine for me because after Ebola I never knew of any sickness prevail was able diagnose me of being ill and also give us food to eat each time we visit and transported us”

—49-year-old female EVD survivor

“I [completed] ended the prevail study, even when I was pregnant I was referred for treatment, the study was good for me”

—28-year-old female EVD survivor

PREVAIL often offered diagnoses of physical and mental ailments caused by EVD that may have been difficult to receive in normal public health facilities. Additionally, PREVAIL offered supplemental support to pregnant women to safely deliver healthy babies, after many experienced recurrent miscarriages just after recovering from Ebola. A WHO 2015 report implies that “[m]ost pregnant woman infected with EVD will lose the unborn child sometime during the course of the disease. Women who recover from EVD with an intact pregnancy (no rupture of membranes or labour) require comprehensive IPC precautions to prevent exposure to intrauterine contents (i.e., amniotic fluid, placenta and fetus) during childbirth or complication management” [71] (p. 15). Thus, having access to specialized prenatal care because of PREVAIL appeared to offer great relief and was deeply appreciated by pregnant survivors.

Additionally, nearly all 19 survivors indicated that they felt like heroes by participating in EVD response work and PREVAIL. They felt a sense of giving back to a greater good. The statistical analysis indicates that, on average, the 19 survivors reported suffering from 5.25 of a total 7 psychosocial stressors (95%CI 4.65–5.85), or 75% aggregated PSS, as a direct result of their EVD status in 2022. The stressors include anger, withdrawal, sadness, poor sleep and eating habits, flashbacks, and anxiety. The most frequent stressors that they reported facing included anger (94.4%, 95%CI 82.7–99.9%), flashbacks (94.1%, 81.6–99.8%), and anxiety (88.2%, 71.2–98.9%). Yet those who felt like Ebola heroes tended to exhibit less PSS (r = −0.54, *p* < 0.001), thus offering support for the positive effects that human participant volunteering can provide. This long-haul stress is elaborated on in a sister publication focused on general experiences of survivors [72]. This finding supports our second hypothesis that Ebola survivors’ participation (when positive) in PREVAIL is linked to improved self-efficacy and psychosocial health. Yet these positive effects appeared to wash out when human participants faced negative PREVAIL incidences.

“Prevail had a good start welcome us with smile and great respect, snacks as breakfast and later lunch as times goes back no snacks was out and we started to overstay… the rumors was about after the five years study we could have benefit (medicine, operations, stipends) …My concern is prevail ended with no benefit for survivors after five years of study”

—40-year-old man

“Prevail has been good… help us to know our health status; my issue is prevail ended the study without any [medical or financial] benefit for survivors”

—28-year-old woman

“[T]he [PREVAIL] study didn’t favor us survivors even when [we went] to protest…since than no action… my eyes are having problems and I told the study but no solution had been taking towards that [when promised]”

—40-year-old male

Comparatively, our analysis of the survey statements indicates a disconnection between what PREVAIL truly provided to participants compared to what the 19 participants were led to believe that they would receive. Most provided statements implying that they were not made adequately aware of their role, mainly as a human participant in biomedical research versus being included as a medical patient, and the benefits that they were guaranteed. Nearly half of the survey participants made statements of not receiving medical treatment beyond an initial diagnosis, and continually going to clinical appointments in anticipation of receiving medical procedures that would cure their ailments, therapy for mental health issues, or provision of free medication.

**Figure 2:**
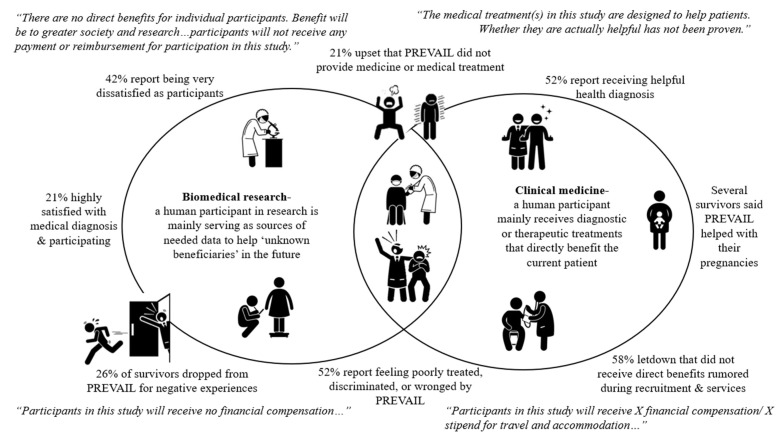
Surveyed Ebola survivors’ aggregate PREVAIL experiences compared to standard IRB participant roles and beneficial and statements in biomedical research verses clinical medicine.

Major health research institutions acknowledge the importance of how adults being able to voluntarily consent may be compromised by certain circumstances including “economic or educational disadvantage, and physical handicap” [73] (paragraph 11). Because the source of the problem seemingly lies in their role as participants, we felt it important to specifically map the differences between biomedical research and clinical medicine. We visually present the confusion that the survey participants presented as experiences against this mapping (see Figure 2).

While clinical trials can offer medical treatment directly to human participants, there is a standard divide between the specific roles and benefits of the human participant that ethical standards repeatedly assert must be stated clearly in ethical approval documents, including IRB consent forms. Typically, clinical medicine holds the patient as the direct beneficiary of the treatment, with the assumption that, while the effects of treatment may still not be known, the intent is that any benefits will impact the wellbeing of the patient. Comparatively, biomedical research implies that the participant is a needed source of medical information, which will not necessarily benefit the participant but offer knowledge benefiting greater society and future vulnerable populations. These distinctions can be noted in commonly standardized IRB consent forms, in which benefits and compensation are specified. For instance, if a participant will or may be randomly selected for treatment, the potential benefits are specified while highlighting the fact that their effect is yet to be scientifically proven [38,73,74,75]. Yet in clinical trials or human subject research with no direct benefits, statements like those presented in the Figure need to be stated [73,75]. Our study, however, finds that one in four of the nineteen participants report dropping out of PREVAIL mainly due to negative experiences.

#### 3.1.2. Lack of Clarity of the Participant Role and Selection for Medical Benefits

A review of PREVAIL documents indicates that they clearly define the role of EVD survivors as study participants, with no mention of them as medical patients [14,15,16,18,28,29,76]. Yet to what extent the 19 survivors understood this role versus what they came to understand it to be (expected or led to believe) appears questionable. Firstly, many of the 19 survivors appeared to have not been made adequately aware of the selection process for some of the randomized trials. Nearly all PREVAIL participants received an initial eye screening, but only specific survivors received more extensive treatment for eye problems [17,77]. Yet in reading the 19 survivors’ comments on eyecare treatment, most expected to have the chance at receiving direct medical treatment like uveitis treatment, as a patient would. Statements like those below indicate a high level of confusion and frustration in the eye examination and treatment selection process.

“[M]y eyes are having problems and I told the study but no solution had been taking towards that”

—36-year-old female

“My concern is prevail took people to the America for eye, since the first group came we have not heard when the other will go America for their eye”

—30-year-old female survivor

It also seems that they did not realize that they were study participants with the sole medical benefit of receiving a diagnosis. There was also confusion among survivors about the selection process for randomized control trials, which appeared to have never been fully cleared up by PREVAIL. Many participants anticipated receiving more robust treatment, including being brought to the United States for uveitis studies, of which only a small number ultimately were, causing disappointment and anger among those not selected. With 30% of survivors with uveitis issues going blind [17,77], we posit that many more PREVAIL participants were just as desperate for medical remedies beyond diagnosis.

Secondly, there are also indications that randomized selection processes were compromised by corrupt practices. There were no prompts for issues of corruption, so this finding was surprising. Survivor statements refer to rumors of being able to bribe a staffer for placement in a treatment group instead of the control. There were also rumors of unethical practices including coercion and sexual exploitation.

“[S]paces [for eye treatment in the US] were sold if you don’t have the money, then you end up having sexual abuse with anyone that is in charge”

—31-year-old female survivor

“[S]urvivors could go America for eye and lost [loss] of membrane, but those spaces were sold…”

—31-year-old female survivor

There must be a clear distinction between gossip and actual reported facts. The reliability and accuracy of data are often based on the quality of the source [59,60]. However, these statements appear to be more than general gossip but instead secondary-sourced evidence. Several survivors reported directly hearing and knowing an individual person who was victimized by these practices but did not formally come forth to report their experience for fear of retribution.

Thirdly, while diagnoses and prescriptions for ailments were provided, typically the program did not cover costs for medical treatment outside of diagnosis, including any needed hospitalization or medication purchases. Instead, prescriptions were provided by PREVAIL medical staff to individual survivors, unless the person was participating in an experimental trial [14,17]. A total of 21% of the 19 individuals reported feeling let down that PREVAIL did not provide medical care, pay for additional medical treatment, or cover medicines for ailments. These statements indicate an additional ethical concern that these provisions were not concretely communicated and assessed by PREVAIL to all the study participants despite their ongoing financial difficulties and obvious desperation for any support with their ongoing physical and mental health complications.

“I started 2016, and the was no benefit for survivors, to the extend that PREVAIL will diagnose a[n] illness and give you the update without a treatment, if you the survivors don’t have means of getting treatment at a hospital, and that could cause death than you made die from that illness that also was the cause of low enrollment at the study”

—31-year-old female survivor

#### 3.1.3. Burden on Study Participants

Initially we hypothesized that participants would receive some financial benefit, such as the provision of stipends and food for attending each clinical appointment, based on reports by PREVAIL. Yet there appears to be heavy transaction costs that fell on some study participants, which appear to be insufficiently addressed by PREVAIL. Most Liberian households are vulnerable to conditions of poverty, and 80% of Liberians have inconsistent work and live on less than USD 1.25/day [78]. All 19 Liberian participants in our study previously had a job before Ebola, yet 7 of the 19 participants (36%) report ongoing issues with unemployment as a direct result of their Ebola status.

“No money. I’m a single mother that hustling to get food for the kids (4) even finding meals at times is difficult for me so means of sending them to school”

—40-year-old female survivor

“No support being a single mother I can only afford meal for the kids not even regularly a week my kids eat, things actually gone bad for my family”

—42-year-old female survivor

PREVAIL reportedly offered varied compensation to participants throughout its different phases. Transportation stipends were provided (around USD 10–20/trip), and, eventually, meals including breakfasts and lunches were also given (at an estimated cost of USD 2/participant per day) [18,29]. But these provisions seemingly changed over time.

Statements like these below imply issues of inadequate or inconsistent financial support and provisions:

“Prevail had a good start welcome us with smile and great respect, snacks as breakfast and later lunch as times goes back no snacks was out”

—40-year-old male survivor

“[W]hen you go to the study no water to even drink and you have to over stay before getting meal”

—36-year-old female survivor

**Figure 3:**
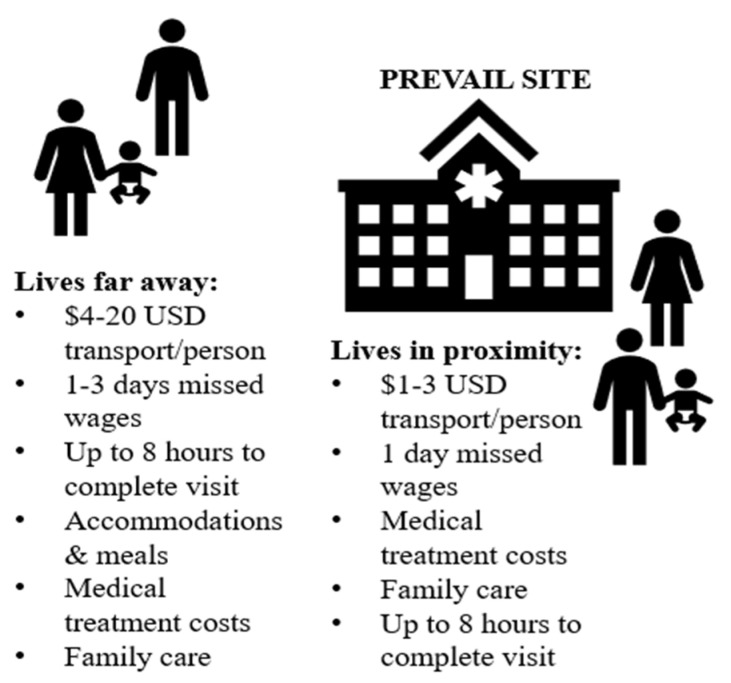
Average transaction costs of Liberian EVD survivors in the PREVAIL study.

The transaction costs reported by the 19 survivors to be able to participate in the PREVAIL study appear extensive, especially for impoverished, single-parent households (see Figure 3). PREVAIL offered travel stipends based on the distance from clinic sites, with guidelines that each visit would require approximately one to two hours per appointment [20,79,80]. For instance, a recruitment ad for PREVAIL III specifies the following:

“Participants will have 1 clinic visit. They will have a physical exam. Their vital signs will be measured. They will also have a neurological checkup. The exam will assess their mental status. Their senses, reflexes, and coordination will be tested. They will be observed while walking to assess their gait. This exam will take about 1 h. Participants will have an interview. They will answer questions about any symptoms they have that may be affecting the brain or nervous system. This will take about 1 h”

[81] (p. 2).

By contrast, nearly all the survivors in our survey stated that the travel stipend was insufficient, as it only paid for participants, and most visits required up to eight hours a day. Based on the estimates that they provided, we gauge that a single clinical visit could have equated up to USD 20 in transportation per person (often needing to travel with child(ren)); one day’s wages for extended examinations over the promised hour time-length; and up to 3 days of wages if transporting from another county, plus accommodations, meals, and potentially family care. PREVAIL’s protocols of time and financial costs to participants appear miscalculated, at least for these survivors.

“I was able to end the study, the study started with snacks morning hours and little lunch follow noon hours, but all changes we arrive by 8:00 am and stay till 4:00 for only lunch than change of attitude of prevail staff towards survivors became too harsh”

—45-year-old female survivor

PREVAIL offered basic financial compensation, which may have contributed to higher stress among participants. Furthermore, nearly all of the 19 survivors made statements reflecting a misunderstanding or poorly communicated information about their contribution being in-kind and not monetarily compensated beyond the general participant stipend. In total, 58% gave statements reflecting sentiments of frustration at perceived broken promises about direct benefits if they completed the full trial(s).

“I heard that after the studies survivors was going to benefit and nothing was giving to us from prevail”

—43-year-old male survivor

“[T]he rumors was the end of prevail study survivors was going to have benefit and that was false”

—42-year-old female survivor

The literature review could find no documentation by PREVAIL identifying these financial issues in its reports.

#### 3.1.4. Unethical Treatment of Study Participants

Another unanticipated implication of this 2022 study was the magnitude of study participants who reported instances of experiencing discriminant or improper treatment by PREVAIL team members. In total, 52% of written testimonies include at least one negative interaction.

“I participate in the study and the beginning was fine but later the staffs started using harsh words on us their interest for survivors decrease and cause dissatisfaction for me”

—35-year-old male survivor

“Prevail never treat me well”

—27-year-old female survivor

“[There was a] change of attitude of prevail staff towards survivors became too harsh”

—45-year-old female survivor

“[P]revail [staff] said we survivors are trouble makers”

—43-year-old male survivor

“I want prevail to come back to tell us about out benefits, because the study didn’t favor us and survivors even when to protest [to the ESNL and MOH]”

—36-year-old female survivor

Statements like these reflect that at first the interactions with PREVAIL staff were pleasant, but as the study continued, the quality of care and treatment of many of the human participants diminished. The next section examines how poor treatment and discriminant experiences impacted their psychosocial health and satisfaction in participating in PREVAIL.

### 3.2. Statistical Analysis

A correlation analysis triangulated many of these key finds. There is a significant negative association between PREVAIL participant satisfaction ranking and receiving insufficient financial compensation (r = −0.51), extensive time requirements for each medical visit (−0.40), and being poorly treated by clinical staff (−0.67) (see Table 3). Medical treatment provision, unequal treatment opportunities, financial insecurity due to disease status, and major demographics appear non-significant. Those who dropped out of PREVAIL tended to have significantly lower PSS levels than those who stayed until the end (r = −0.45, *p* < 0.05).

There were approximately 1500 Liberian EVD participants and 6000 close contacts including family members who participated in PREVAIL’s various trials. Our study’s small sample size (n = 19) limits the causal generalizability of findings to larger populations. Thus, we addressed this statistical limitation by applying bootstrap regression modeling. We noted that the modeling appeared significant (replications = 999). We conducted nonparametric bootstrapping that involved randomized sampling with replacement. This method excludes any assumptions concerning underlying population distribution. It serves as an alternative for hypothesis-testing in small-N studies. By conducting bootstrap regression modeling, we could assess the model parameters’ variability and, more so, the extent of random variation within each coefficient related to incremental changes in data values [82,83,84].

Despite the limited and non-randomized sampling, bootstrap modeling indicated that some findings might be statistically significant. In Table 4, if a survivor experienced poor treatment as a study participant by PREVAIL staff, their overall satisfaction would predictably be 31.67% percent lower (0.95 points out of a total of 3 (high)) than peers who did not (*p* < 0.001). Yet the effects of feeling they had unequal access to additional medical services like sight treatment, along with experiencing long clinic visits, were limited (*p* > 0.05).

Moreover, the EVD survivors self-reported their stress symptoms that they continued to experience as a direct result of their disease status five years after recovery. An additional bootstrap model indicated that those who stated experiencing an incident of being poorly treated or feeling discriminated by PREVAIL staff appeared to have an 18 percent (1.27 points) higher rate of psychosocial stress (SE = 0.63, bias = 0.10, normal CI (0.05–2.50); number of observations = 13; replications = 632, Adj. R^2^ = 0.59, Wald χ^2^ = 66.48, *p* = 0.00).

## 4. Discussion

Both the qualitative analysis of key literature review findings and the open-ended responses to survey questions, along with the statistical analysis, imply that survivors who participated in PREVAIL experienced some benefits, at least initially. The survivors reported benefiting from free medical diagnoses and an initial sense of purpose for the greater good, including feeling like heroes. However, we question if the benefits of participating in medical trials outweigh the burdens and negative experiences that appear to be commonly shared experiences. The anticipated benefits of participating, often informed by their dire impoverished state and physical impairments because of surviving EVD, were not consistently met. The transaction costs often negatively impacted the survivors’ experiences. Likewise, there appear to be critical issues concerning the specific benefits survivors could expect, along with the treatment that they would receive, and what was provided. Often, it seemed to be an issue of the “carrot on the stick”, in which misinterpretations of the roles of participants as human subjects rather than medical patients went unchecked, and inconsistent messaging led to rumors of corruption. The participants mostly appeared to have had a positive experience in the beginning of their engagement in PREVAIL, but, over time, their experiences in general worsened. The treatment of participants diminished over time, leading some to experience significantly more psychosocial stress and negative feelings like resentment.

The experiences shared by these 19 survivors provide some initial insights into PREVAIL’s operations, as well as the meaning of hardship among study participants as a shared experience. The data from our literature review of 331 sources, combined with the mixed method analysis of the survey data from the 19 Ebola survivors, indicate that this disease-affected population was likely highly vulnerable before PREVAIL was launched. Such scenarios can probably inform contextual vulnerability that can impair a person’s ability to fully consent or fully understand the details of clinical research participation without being influenced by these negative factors [6,18,34,37]. Any medical research initiative engaging in recruitment of a vulnerable population should consider how to address their vulnerabilities, including monitoring and evaluating potential unanticipated implementation problems [27,28,37,38].

There are some important factors that the literature review reveals about PREVAIL’s ethical preparedness for working with survivors. PREVAIL’s timeline appears hurried for protocol development and launch. For example, PREVAIL II’s protocol development started in late November 2014, with NIH IRB approval granted on 29 January 2015, the approval of the Liberia National Research Ethics Board (NREB) granted on 13 February, and the “first patient” (only document referring to EVD survivors with this term) examined on 22 March [18]. Standard ethical approval for clinical trials by ethics institutions tend to be longer [37,39,47,56]. The NIH’s digital instructions for IRB approval of clinical trials with human participants in international countries “often take a long time” [22]. Moreover, a key document reveals that “there was a great deal of disagreement among researchers over how clinical trials should be designed during the Ebola epidemic, particularly over whether trials should use randomization and concurrent control groups” [57]. PREVAIL was launched after eventual consensus was reached that it would be unethical to deprive patients of a treatment that could possibly prevent or treat Ebola.

PREVAIL studies and documents mention very few of the vulnerabilities that survivors faced in relation to ethical considerations. Instead, most documents include diction like “rapidly appraise”, “making progress”, and “rapid ethics and regulatory review” [18,20,31,32], possibly reflecting a lack of proper attention before implementation. In general, satisfaction with PREVAIL varied among the 19 survivors, statistically linked to implementation issues like being treated poorly by staff. While some volunteered to help inform the understanding of EVD and to benefit from medical diagnoses, in general most were motivated by desperation for medical service provision, mainly in the hope of some relief from their physical, mental, and socio-economic hardships [6,9,28,34,58]. We do not know the extent to which PREVAIL monitored and evaluated the experiences of its study participants. The comments indicate that at some point, EVD survivors formally protested issues of dissatisfaction, compensation, and medical treatment to GOL and ESNL. Ethical protocols in any EVD-related program that assess for potential negative unintended consequences could play a vital role in better safeguarding participants [6,26,28,59]. As our study implies, EVD survivor participant experiences with recruitment, retention, and treatment varied widely. We posit that implementation standards may have been too loose and inconsistent throughout the different PREVAIL trials, leading these 19 EVD survivors, at least, to feel confused about benefits, their roles as participants, and what to expect.

The survivors’ feedback highlights additional vulnerabilities because of their experiences with PREVAIL. Employment with PREVAIL was promised, but most of the participants remarked that few survivors were hired. Stipends and the provision of meals were inconsistent, and there was the likelihood that participating in PREVAIL economically burdened many households. Survivors faced added forms of social vulnerability, as well as deferential vulnerability, in that many of them felt discriminated against, poorly treated by, or lied to by PREVAIL staff, likely damaging their emotional health.

Likewise, their medical vulnerability due to ongoing health issues and the institutional vulnerability of a sick population being targeted repeatedly to volunteer for the study further highlight ethical concerns. Many survivor testimonies reflect the participants’ impressions that PREVAIL was designed to provide medical support (even treatment) that would help their own physical health ailments. Only a few appeared to have understood that while there might have been a randomized chance to receive specific treatments like eyecare or vaccines, for the most part, their roles were as sources of data on Ebola. Their blood, statements, and physical exams would inform research contributing mainly to generating collective knowledge that would benefit others in the future. It is questionable to suppose that patients mainly enroll in medical trials solely to support advancing collective knowledge and the benefit of future generations, especially when facing life-threatening diseases. Most EVD survivors were motivated by their personal needs and wellbeing, not altruism. The survivor participants appeared to have felt coerced to participate in the hopes of receiving a direct benefit if they completed the trial. Some indicated that there were issues of corruption and abuse in their dealings with PREVAIL staff, including bribes and trading sexual favors to be placed on prioritization lists for care.

As Salhia and Olaiyaand (2020) indicate, “[t]oday, clinical trials are one of the most heavily regulated practices in the world, and yet still not all people are provided the same oversights and protections, with improprieties disproportionately affecting poor-resource nations and vulnerable populations” [1] (paragraph 1). The ethical hardships that the survey participants report facing as PREVAIL participants highlights the need for the global health community to revisit how we conduct biomedical research in low-income countries, where participants are first made vulnerable due to impoverished circumstances and a lack of healthcare access and then see these compounded by the negative life factors and health impairments that they face after surviving a deadly disease. The NIH and other federal agencies are obligated to adhere to standards of conduct and ethical standards prominent in Western research cultures. Yet high ethical standards and practices can be compromised or hard to ensure in crisis environments, as demands tend to be intense, with limited resourcing, and thus “corners are cut” [3] (paragraph 13). The same factors may also linger even in post-outbreak and recovery phases, living clinical trials open to issues of ethically protecting highly vulnerable research participants in regions like Africa.

### Limitations

This study applied a mixed methods approach, merging quantitative analysis with the qualitative exploration of survivors’ experiences and the intrinsic meaning behind their experiences as human participants. The main objective of collecting open-ended answers was to capture narratives that explore the essence behind their individual EVD-related experiences [59,60,85]. The generalizability of the statistical findings is limited by the small sample size. This research study (like other small-N studies) tended to provide simplified statistical analyses, establishing correlation patterns but not necessarily causal mechanisms beyond associated relationships. [86,87,88]. However, as we discussed above, bootstrapping may better inform generalizability, but this has its limitations [59,82,84,85,89]. Future larger-scale and/or randomized control trial studies may better inform the generalizability of these results to the general EVD population.

## 5. Conclusions

This research reminds the health field of the importance of the unique contextual factors and ethical risks that medical research can have on human participants when implemented in low-income settings beset by poverty-based vulnerabilities. Medical professionals and partners must carefully weigh the benefits of the research against the burdens on participants, many of whom live in extreme poverty and are marginalized due to their disease status. Medical research on hemorrhagic diseases like Ebola plays a critical role in providing deeper understanding of less-known contagions, life-saving vaccinations, and opportunities for those affected by the disease to benefit through participation. PREVAIL led to massive gains in medical knowledge not previously afforded, including vaccination against EVD. PREVAIL also offered survivors targeted medical evaluations and individualized care. Yet the burdens for many EVD survivor participants appear to have outweighed the benefits, leading to increased psychosocial stress, financial hardships, and, in some cases, participant mistreatment.

Ebola biomedical research garnered large international support around the outbreak, including significant increases in funding. Yet when engaging a vulnerable disease-affected population, research must be cautious against rushing the ethical approval process and set up proper measures to safeguard study participants. Likewise, clinical research in low-income settings like Liberia must be careful to clearly communicate the roles, benefits, and processes that participants will undergo, to avoid needless confusion and the sense of being misled. Research and medical interventions like PREVAIL may have been designed with good intentions but were ultimately poorly implemented and at times did not prove adequate to fulfill survivors’ needs. The sheer volume of vulnerabilities EVD survivors endured after discharge from ETUs should have served as a clear warning to PREVAIL of participants’ desperation to seek any potential solutions to their problems—medically or otherwise. In these circumstances, health organizations must take precautions to clearly understand, articulate, and communicate the different roles, benefits, and needs of participants of vulnerable populations in biomedical research verses medical interventions. They must also consider putting in place frameworks that monitor and safeguard against ethical misconduct and undue burdens on survivor participants, many of whom may have less awareness and resources to protect them.

## Figures and Tables

**Table 1:** Types of vulnerability compared to EVD survivors’ quality-of-life factors.

Type of Vulnerability	Relation to EVD Survivors’ Quality of Life
Medical	EVD survivors continue to struggle with long-term poor psychosocial health, which increases over time [12]. Long-haul physical EVD complications include chronic headaches, cognitive impairment, body aches, impotency, and infertility. In total, 1 in 100 Liberians had regular access to mental health services [5,23]
Economic	Most survivors lost their jobs or a household breadwinner due to the disease [5,41,61,62].
Social	Many female and male survivors were physically attacked, beaten, emotionally abused, shunned, illegally convicted, and labeled by others as tainted, dangerous, or victims of ill fortune [23,29,63,64]
Cognitive	75–90% of EVD survivors experienced complications for several years after primary symptoms were resolved [64,65]. EVD-related cognitive, psychiatric, and physical abnormalities led to additional PREVAIL studies [22,66]
Institutional	Liberia tends to be a society valuing both formal and informal hierarchical structures, including community elders and traditional leaders with heavy influence over the lives of people in their ethnic groups [67,68]. Nearly half of adult Liberians would prioritize supporting the decision of traditional leaders in decision making [69]. Many traditional elders often stigmatized and evicted EVD survivors from their communities for fear of contagion spread or bad juju affecting others [23,70]
Deferential	Informal and formal hierarchies that create institutional and deferential vulnerabilities can limit a participant’s true autonomous decision making and place them at risk of exploitation. In low-income settings with limited healthcare systems, patients have less choice or voice in medical care due to high demand and low supply. The Liberia Medical and Dental Council (2016) has stated that Liberia’s medical and health services are low-resourced, and after EVD, there were 298 medical doctors responsible for the national population (doctor-per-patient ratio of 1:15,000)

**Table 2:** Summary results of key areas of interest.

Area of Interest	Summary Results
Long-haul EVD effects	Less child stigmatization; positive feelings of being heroes; high psychosocial stress; long-term EVD physical impairments; unemployment and work and discrimination due to disease status; difficulty receiving medical treatment in public facilities; undervalued social group
Participant experience with PREVAIL	High satisfaction with medical diagnoses provided; medical and compensation benefits for human subjects not clearly established, causing confusion and frustration; common misunderstanding of the trial selection process; diagnosed with medical conditions but many left under the impression that they would receive medical treatment to improve their help; issues with what was promised in the recruitment process
Burden on human participant	Heavy transaction costs falling on impoverished human subjects; issues of inadequate or inconsistent financial support and provisions; time costs to participants appearing greatly miscalculated (estimated 8 h long visits plus transportation time)
Unethical treatment of human subjects	Instances of discriminant or improper treatment by PREVAIL team members; 52% of survey testimonies include at least one negative experience; rumors of unethical practices including coercion and sexual assault
Statistical implications	Significant negative association between PREVAIL participant satisfaction ranking and receiving insufficient financial compensation (r = −0.51), extensive time requirements for each medical visit (−0.40), and being poorly treated by clinical staff (−0.67)

**Table 3:** Point-biserial correlation analysis of PREVAIL experiences among Ebola survivors.

	No Medicine Provision	No Financial Compensation	Treated Poorly by Staff	Long Visits (around 8 h)	Unequal Treatment Opportunities
Satisfaction with PREVAIL (1 low, 3 high)	−0.36	−0.51 **	−0.67 ***	−0.40 *	−0.18
Financial insecurity due to Ebola	−0.13	−0.45 **	−0.15	−0.04	−0.21
Sex (female 1, male 2)	−0.31	−0.21	0.09	−0.15	−0.27
County (Montserrado 1, Margibi 2)	−0.17	−0.05	−0.02	−0.23	0.05

Note: * *p* < 0.05, ** *p* < 0.01, *** *p* < 0.001.

**Table 4:** Statistics for bootstrapped regression modeling of 2022 Liberian EVD survivors’ satisfaction as study participants in PREVAIL.

		Coefficient		
	Constant	Poor Treatment by PREVAIL Staff (1—yes; 0—no)	Unreasonably Long Clinic Visits (1—yes; 0—no)	Unequal Medical Treatment Access (1—yes; 0—no)
Avg. bootstrap estimate	2.43 ***	−0.95 ***	−0.48	0.31
Bootstrap SE	0.22	0.30	0.42	0.36
Bias	0.02	−0.04	0.01	−0.02
Normal CI	(1.99–2.87)	(−1.54–−0.36)	(−1.31–0.34)	(−0.65–0.72)
Percentile CI	(1.98–2.87)	(−1.56–−0.35)	(−1.25–0.36)	(−0.62–0.74)
Bias-corrected CI	(1.85–2.80)	(−1.44–−0.27)	(−1.26–0.32)	(−0.63–0.74)

Notes: Conducting nonparametric bootstrapping involves randomized sampling with replacement, excluding any assumptions concerning underlying population distribution; serves as an alternative for hypothesis testing in small-N studies; and involves assessing the model parameters’ variability and extent of random variation within each coefficient related to incremental changes in data values [82,83,84]. Two bootstrap confidence intervals (CIs) are shown for each coefficient. Standard error = SE. *** *p* < 0.01. Number of observations = 19; replications = 999. Adj. R^2^ = 0.43, Wald χ^2^ = 19.11, *p* = 0.00.

## Data Availability

The datasets presented in this article are not readily available because of ethical requirements of the IRB to safeguard participant anonymity. Requests to access the datasets should be directed to jhansonde@ltu.edu.

## Appendix A. Semi-Structured Survey

Q1 Do you consent to participate in this study? Yes, I consent (1); No, I do not (0)

Q2 Have you ever had Ebola? Yes (1) No (0)

Q3 How old are you? (participants must be between 18–65)

Q4 Where do you currently live? (county name)

Q5 What is your gender? Male (1) Female (2) Other (3)

Q6 When did you have (first get infected with) Ebola? What month and year?

Q7 Do you continue to suffer today from any of the following symptoms or negative experiences because of surviving (having) Ebola? (Yes-1, No-0 for all below—Anger, withdrawal, sleeping issues, eating problems (eating too much or too little), anxiety, sadness (sad most days of the week), flashbacks, stigma from others, financial issues or not enough work

Q8 Did you receive assistance (stipend, food stuff, etc.) after being discharged from the ETU? Yes (1) No (0)

Q9 What years did you receive this assistance? Select all years that apply: 2014–2016 (1); 2017–2019 (2), 2020–2021 (3), today (4)

Q10 How satisfied were you with this assistance, on a scale of unhappy face (not at all 1) to smily face (extremely satisfied 5)?

Q11 Did you and/or a family member in your household participate in PREVAIL (Partnership for Resaerch on Ebola Vaccines in Liberia) study? If so, what years? 2014–2017

Q12 (a) If so, did you ever stop participating? Yes (1) No (0) (b) Why? (please explain your experiences with the study, the program, and the quality of care, and any issues you had)

Q13 Did you hear any rumors about PREVAIL that concerned you? Yes (1) No (0), if so, what were the rumors?

Q14 Did you have any other concerns of comments about the PREVAIL study? Yes (1) No (0) Explain

Q15 How much has the government policies supporting Ebola survivors helped you, or not? Did not help at all (1) Somewhat helped (2) Helped a lot (3) Explain

Q16 Were there any promised policies programs for Ebola survivors that were not offered or the quality was very poor? Please explain

Q17 What assistance do you need most today because of being an Ebola survivor?

Q18 Click on the statements that you agree with: I feel like the world has forgotten about Ebola survivors (1); Ebola has changed my life forever (2); Being an Ebola survivor makes me a hero (3)

Q19 Do you have any biological/born or adopted children you care for? Yes (1) No (0)

Q20 If you have children you are responsible for, do they currently go to school? Yes (1) No (0)

Q21 If yes, Is the student welcomed and treated fairly, or do they face issues with stigmatization because they come from an Ebola-affected home?

Q22 If you have kids who are not going to schook, what are the reasons they are not in school?

Q23 Were you working before you got sick with Ebola? Yes (1) No (0)

Q24 Have you had any trouble finding work since, because you are an Ebola survivor? Yes (1) No (0)

Q25 Please explain your work experiences since Ebola ended.

## Appendix B

**Table A1:** PREVAIL Staged Studies and Contributions.

**PREVAIL Study**	**Key Examples of Reported Results**
PREVAIL I Double-blind, placebo-controlled RCT of an Ebola vaccine	Among 650 close contacts and contacts of close contacts of EVD cases, 210 (32%) consented and were vaccinated with rVSVAG-ZEBOV-GP. Of those vaccinated, 189 (90%) attended the 1-month follow-up visit; 166 (79%) attended the 6-month visit. No serious adverse events were reported. Among 88 participants without an elevated antibody level at baseline, 77.3% (95% confidence interval, 68.5–86.1) had an antibody response at 1 month [88]
PREVAIL II RCT of the experimental treatment ZMapp	Of the 71 patients who could be evaluated, 21 died, representing an overall case fatality rate of 30%. Death occurred in 13 of 35 patients (37%) who received the current standard of care alone and in 8 of 36 patients (22%) who received the current standard of care plus ZMapp; although the estimated effect of ZMapp appeared to be beneficial, the result did not meet the prespecified statistical threshold for efficacy [85]
PREVAIL III Observational natural history study of Ebola survivors and their close contacts	The stigma questions were administered to 859 EVD survivors at initial visit and 741 (86%) survivors at follow-up. While 63% of survivors reported any stigma at the initial visit, only 5% reported any stigma at follow-up. Over the 18-month period, there was a significant decrease in stigma among EVD survivors (Adjusted Odds Ratio [AOR], 0.02; 95% Confidence Interval [CI], 0.01–0.04) [89]
PREVAIL III Sub-studies: Birth cohort Evaluation of children born to EVD-survivor participants CYTOF Detailed immunological analysis in select survivors and close contacts; Neurology Longitudinal follow-up study of neuropsychological sequelae of EVD; Ophthalmology Longitudinal ophthalmologic (eyecare) follow-up study of a subset of survivors and close contacts; Viral persistence in semen; Assessment of the persistence of Ebola virus in the semen of adult male participants after acute illness Viral persistence in the CNS Assessment of the persistence of Ebola virus in the central nervous system (CNS) of adult participants after resolution of the acute illness	“Most pregnant woman infected with EVD will lose the unborn child sometime during the course of the disease. Women who recover from EVD with an intact pregnancy (no rupture of membranes or labour) require comprehensive IPC precautions to prevent exposure to intrauterine contents (i.e., amniotic fluid, placenta and fetus) during childbirth or complication management” [71] (p. 15). “Neurologic abnormalities following Ebola virus disease involved subcortical structures, cerebellar pathways, and sensory peripheral nerves and were present in most survivors” [90] (p. 2). Recruitment Ad—PREVAIL III: “Participants will have 1 clinic visit. They will have a physical exam. Their vital signs will be measured. They will also have a neurological checkup. The exam will assess their mental status. Their senses, reflexes, and coordination will be tested. They will be observed while walking to assess their gait. This exam will take about 1 h. Participants will have an interview. They will answer questions about any symptoms they have that may be affecting the brain or nervous system. This will take about 1 h” [78] (para. 5). “Signs of uveitis were present in at least 24% of Ebola survivors…Optic nerve changes, such as nerve swelling, were present in 10% of survivors and 5% of controls (*p* = 0.03), with color vision deficits occurring in 39% of survivors vs. 13% of controls (*p* < 0.001)” [91] (para. 3).
PREVAIL IV Double-blind, two-phase, two-arm trial RCT of GS-5734 among male Ebola survivors with persistent Ebola virus RNA in their semen	“38 men from July 2016 through June 2018. The mean treatment phase ANRs were 85% (standard deviation [SD] = 24%) and 76% (SD = 30%) in the remdesivir and placebo arms, respectively (*p* = 0.270). The mean follow-up phase ANRs were 96% (SD = 10%) and 81% (SD = 29%) in the remdesivir and placebo arms, respectively (*p* = 0.041). The 5-day remdesivir regimen was well tolerated with no safety concerns… this small trial, remdesivir 100 mg/day for 5 days safely reduced the presence of Ebola virus RNA in the semen of Ebola survivors 2 to 6 months after administration” [16] (para. 3-4).
PREVAIL V (PREVAC) Quadruple blind, placebo-controlled RCT of two Ebola vaccines	“Of the 4789 participants, 2560 (53%) were adults and 2229 (47%) were children. Those < 18 years of age included 549 (12%) aged 1 to 4 years, 750 (16%) 5 to 11 years, and 930 (19%) aged 12–17 years. At baseline, the median (25th, 75th percentile) antibody titer to Ebola virus glycoprotein for 1090 participants was 72 (50, 116) EU/mL” [22] (p. 2). (Involves VSVdeltaG-ZEBOV vaccine and ChAd3-EBO Z vaccine and placebo (later given option to have treatment))
PREVAIL VI Observational study of the genetic factors underlying Ebola risk and response	Some participants will have their blood tested for the infection syphilis and HIV, the virus that causes AIDS. Participants will be told the results and will receive help finding care, if necessary. “The death rate among those with HIV was 12.3% compared to 1.9% in HIV-negative individuals (adjusted odds ratio of 6.87)” [92] (para. 3).
PREVAIL VII Clinical trial to evaluate the persistence of Ebola viral RNA in the eyes and assess the response to cataract surgery of Ebola survivors	“Twenty-two eyes of 22 survivors and 12 eyes of eight controls underwent cataract surgery. All of the aqueous samples tested negative for Ebola viral RNA. Median visual acuity improved from 20/200 at baseline to 20/25 at 1 year in survivors and from count fingers to 20/50 in controls (overall, *p* < 0.001; between groups, *p* = 0.07). After a 1-month course of topical corticosteroids, 55% of survivors and 67% of controls demonstrated at least 1+ anterior chamber cell. Twelve months after surgery, optical coherence tomography revealed a median increase in macular central subfield thickness of 42 µm compared with baseline (overall, *p* = 0.029; between groups, *p* = 0.995) EVD survivors and controls demonstrated significant visual improvement from cataract surgery” [17] (para. 3).

## References

[1] Salhia B., Olaiya V. (2020). Historical Perspectives on Ethical and Regulatory Aspects of Human Participants Research: Implications for Oncology Clinical Trials in Africa. JCO Glob. Oncol..

[2] Hanson-DeFusco J., Shi M., Du Z., DeFusco A. Conditioned Learning of Nations-Influence of EVD-Related System Adaptations and COVID-19 Response. Harvard Dataverse: 2022.

[3] Darrow W.W. (2023). Correction to: Confronting AIDS in the Early 1980s: Biomedicine, Public Health, and the Fourth Estate. AIDS Behav..

[4] Perry K.E., Dubé K., Concha-Garcia S., Patel H., Kaytes A., Taylor J., Javadi S.S., Mathur K., Lo M., Brown B. (2020). “My Death Will Not [Be] in Vain”: Testimonials from Last Gift Rapid Research Autopsy Study Participants Living with HIV at the End of Life. AIDS Res. Hum. Retroviruses.

[5] Decosimo C.A., Hanson J., Quinn M., Badu P., Smith E.G. (2019). Playing to live: Outcome evaluation of a community-based psychosocial expressive arts program for children during the Liberian Ebola epidemic. Glob. Ment. Health.

[6] Swanson K.C., Altare C., Wesseh C.S., Nyenswah T., Ahmed T., Eyal N., Hamblion E.L., Lessler J., Peters D.H., Altmann M. (2018). Contact tracing performance during the Ebola epidemic in Liberia, 2014-2015. PLoS Negl. Trop Dis..

[7] Matanock A., Arwady M.A., Ayscue P., Forrester J.D., Gaddis B., Hunter J.C., Monroe B., Pillai S.K., Reed C., Schafer I.J. (2014). Ebola virus disease cases among health care workers not working in Ebola treatment units—Liberia, June–August, 2014. MMWR Morb. Mortal. Wkly. Rep..

[8] Pillai S.K., Nyenswah T., Rouse E., Arwady M.A., Forrester J.D., Hunter J.C., Matanock A., Ayscue P., Monroe B., Schafer I.J. (2014). Developing an incident management system to support Ebola response—Liberia, July–August 2014. MMWR Morb. Mortal. Wkly. Rep..

[9] Department of Global Communications (2020). Learning from the Past: UN Draws Lessons from Ebola, Other Crises to Fight COVID-19. https://www.un.org/en/coronavirus/learning-past-un-draws-lessons-ebola-other-crises-fight-covid-19.

[10] Routh J. (2015). Study of Ebola Survivors Opens in Liberia-Trial to Examine Long-Term Health Effects of Ebola Virus Disease. https://www.nih.gov/news-events/news-releases/study-ebola-survivors-opens-liberia.

[11] James P.B., Wardle J., Steel A., Adams J. (2019). Post-Ebola psychosocial experiences and coping mechanisms among Ebola survivors: A systematic review. Trop. Med. Int. Health.

[12] Secor A., Macauley R., Stan L., Kagone M., Sidikiba S., Sow S., Aronovich D., Litvin K., Davis N., Alva S. (2020). Mental health among Ebola survivors in Liberia, Sierra Leone and Guinea: Results from a cross-sectional study. BMJ Open.

[13] NIAID (2022). Partnership for Research on Ebola Virus (PREVAIL) Prevail III: Ebola Natural History Study. https://clinicaltrials.gov/ct2/show/NCT02431923.

[14] Dodd L.E., Proschan M.A., Neuhaus J., Koopmeiners J.S., Neaton J., Beigel J.D., Barrett K., Lane H.C., Davey R.T. (2016). Design of a Randomized Controlled Trial for Ebola Virus Disease Medical Countermeasures: PREVAIL II, the Ebola MCM Study. J. Infect. Dis..

[15] Browne S., Carter T., Eckes R., Grandits G., Johnson M., Moore I., McNay L. (2018). A review of strategies used to retain participants in clinical research during an infectious disease outbreak: The PREVAIL I Ebola vaccine trial experience. Contemp. Clin. Trials Commun..

[16] Higgs E.S., Gayedyu-Dennis D., Fischer W.A., Nason M., Reilly C., Beavogui A.H., Aboulhab J., Nordwall J., Lobbo P., Wachekwa I. (2021). PREVAIL IV: A Randomized, Double-Blind, 2-Phase, Phase 2 Trial of Remdesivir vs Placebo for Reduction of Ebola Virus RNA in the Semen of Male Survivors. Clin. Infect. Dis..

[17] Eghrari A.O., Shantha J.G., Ross R.D., Van Ryn C., Crozier I., Hayek B., Gradin D., Roberts B., Prakalapakorn S.G., Amegashie F. (2021). Efficacy and Safety Outcomes of Cataract Surgery in Survivors of Ebola Virus Disease: 12-Month Results From the PREVAIL VII Study. Transl. Vis. Sci. Technol..

[18] FHI Clinical (2022). Partnership for Research on Vaccines and Infectious Diseases in Liberia (PREVAIL)-Brochure. https://www.fhiclinical.com/wp-content/uploads/2020/08/Brochure_PREVAIL_20200812_SHARE-2.pdf.

[19] NIAID (2022). Research with Human Subjects. https://www.niaid.nih.gov/grants-contracts/human-subjects.

[20] NIAID (2022). NCT02818582 GS-5734 to Assess the Antiviral Activity, Longer-Term Clearance of Ebola Virus, and Safety in Male Ebola Survivors with Evidence of Ebola Virus Persistence in Semen. NCT02818582.

[21] Nyenswah T., Fallah M., Sieh S., Kollie K., Badio M., Gray A., Dilah P., Shannon M., Duwor S. (2015). Controlling the last known cluster of Ebola virus disease—Liberia, January–February 2015. MMWR Morb. Mortal Wkly. Rep..

[22] Badio M., Lhomme E., Kieh M., Beavogui A.H., Kennedy S.B., Doumbia S., Leigh B., Sow S.O., Diallo A., Fusco D. (2021). Partnership for Research on Ebola VACcination (PREVAC): Protocol of a randomized, double-blind, placebo-controlled phase 2 clinical trial evaluating three vaccine strategies against Ebola in healthy volunteers in four West African countries. Trials.

[23] Glayweon M., Hanson J. (2015). Ebola Survivors and Their Needs-Survey Assessment.

[24] CDC (2016). CDC Releases Detailed History of the 2014–2016 Ebola Response in MMWR. https://www.cdc.gov/media/releases/2016/p0707-history-ebola-response.html.

[25] Partner Coordination Workshop for the Support of Survivors (2015). National Package for Resettlement of All EVD Survivors.

[26] Bah A.J., James P.B., Bah N., Sesay A.B., Sevalie S., Kanu J.S. (2020). Prevalence of anxiety, depression and post-traumatic stress disorder among Ebola survivors in northern Sierra Leone: A cross-sectional study. BMC Public Health.

[27] MOH/IMS (2014). MOH/IMS Meeting Summary Notes 9/24/2014-5/5/2015. Ebola IMS Pillar.

[28] Faley P. (2022). Fixing the PREVAIL Ebola project. Proceedings of the UnwokeID: Unethical Issues in 21st Century International Development.

[29] Davis D. (2022). Unequal treatment of Ebola survivors in Liberia. Proceedings of the UnwokeID: Unethical Issues in 21st Century International Development.

[30] Schwartz D.A. (2019). All the mothers are dead: Ebola’s chilling effects on the young women of one Liberian town named Joe Blow. Pregnant in the Time of Ebola.

[31] Krug E.G., Mercy J.A., Dahlberg L.L., Zwi A.B. (2002). The world report on violence and health. Lancet.

[32] Mapitsa C.B., Ngwato T.P. (2020). Rooting Evaluation Guidelines in Relational Ethics: Lessons From Africa. Am. J. Eval..

[33] Astroulakis N. (2011). The development ethics approach to international development. Int. J. Dev. Issues.

[34] Calain P. (2018). The Ebola clinical trials: A precedent for research ethics in disasters. J. Med. Ethics.

[35] Sutton L.B., Erlen J.A., Glad J.M., Siminoff L.A. (2003). Recruiting vulnerable populations for research: Revisiting the ethical issues. J. Prof. Nurs..

[36] Gordon B.G. (2020). Vulnerability in Research: Basic Ethical Concepts and General Approach to Review. Ochsner. J..

[37] Public Responsibility in Medicine and Research (2014). Scientific, Regulatory, and Ethical Issues with Experimental Treatments and Ebola Vaccines. https://www.youtube.com/watch?v=3cf31hS7tKM.

[38] Ministry of Health (MOH) (2015). Mapping of Agencies Supporting Survivors in Response to Ebola.

[39] Hanson J., Decosimo A., Quinn M. (2016). Diminished Quality of Life among Women affected by Ebola. J. Soc. Behav. Health Sci..

[40] Routh J. (2019). Study Finds Ebola Survivors in Liberia Face Ongoing Health Issues. https://www.nih.gov/news-events/news-releases/study-finds-ebola-survivors-liberia-face-ongoing-health-issues.

[41] US National Bioethics Advisory Commission (2001). Ethical and Policy Issues in International Research: Clinical Trials in Developing Countries.

[42] Foa E.B., Street G.P. (2001). Women and traumatic events. J. Clin. Psychiatry.

[43] Hembree E.A., Foa E.B. (2000). Posttraumatic stress disorder: Psychological factors and psychosocial interventions. J. Clin. Psychiatry.

[44] Foa E.B., Riggs D.S., Dancu C.V., Rothbaum B.O. (1993). PTSD Symptom Scale-Interview (PSS-I). https://link.springer.com/article/10.1007/BF00974317.

[45] Chambers M., Bliss K., Rambur B. (2020). Recruiting Research Participants via Traditional Snowball vs Facebook Advertisements and a Website. West. J. Nurs. Res..

[46] Leighton K., Kardong-Edgren S., Schneidereith T., Foisy-Doll C. (2021). Using Social Media and Snowball Sampling as an Alternative Recruitment Strategy for Research. Clin. Simul. Nurs..

[47] Dusek G., Yurova Y., Ruppel C.P. (2015). Using social media and targeted snowball sampling to survey a hard-to-reach population: A case study. Int. J. Dr. Stud..

[48] Ellis J., Hart D. (2023). Strengthening the Choice for a Generic Qualitative Research Design. The Qualitative Report.

[49] Kahlke R.M. (2014). Generic Qualitative Approaches: Pitfalls and Benefits of Methodological Mixology. Int. J. Qual. Methods.

[50] Percy W., Kostere K., Kostere S. (2015). Generic qualitative research in psychology. Qual. Rev..

[51] Caelli K., Ray L., Mill J. (2003). ‘Clear as Mud’: Toward Greater Clarity in Generic Qualitative Research. Int. J. Qual. Methods.

[52] Merriam S.B., Tisdell E.J. (2009). Qualitative Research: A Guide to Design and Implementation.

[53] Lim J. (2011). Qualitative methods in adult development and learning: Theoretical traditions, current practices, and emerging horizons. The Oxford Handbook of Reciprocal Adult Development and Learning.

[54] Gough B., Lyons A. (2016). The Future of Qualitative Research in Psychology: Accentuating the Positive. Integr. Psychol. Behav. Sci..

[55] Ivankova N.V., Creswell J.W., Stick S.L. (2006). Using Mixed-Methods Sequential Explanatory Design: From Theory to Practice. Field Methods.

[56] Creswell J.W. (2004). Designing A Mixed Methods Study In Primary Care. Ann. Fam. Med..

[57] Shadish W.R., Cook T.D., Campbell D.T. (2002). Experimental and Quasi-Experimental Designs for Generalized Causal Inference.

[58] Dunn W.N. (2017). Public Policy Analysis.

[59] Calnan M., Gadsby E.W., Kondé M.K., Diallo A., Rossman J.S. (2018). The Response to and Impact of the Ebola Epidemic: Towards an Agenda for Interdisciplinary Research. Int. J. Health Policy Manag..

[60] Van Bortel T., Basnayake A., Wurie F., Jambai M., Koroma A.S., Muana A.T., Hann K., Eaton J., Martin S., Nellums L.B. (2016). Psychosocial effects of an Ebola outbreak at individual, community and international levels. Bull. World Health Organ..

[61] Davtyan M., Brown B., Folayan M.O. (2014). Addressing Ebola-related Stigma: Lessons Learned from HIV/AIDS. Glob. Health Action.

[62] Tozay S., Fischer W.A., Wohl D.A., Kilpatrick K., Zou F., Reeves E., Pewu K., DeMarco J., Loftis A.J., King K. (2020). Long-term Complications of Ebola Virus Disease: Prevalence and Predictors of Major Symptoms and the Role of Inflammation. Clin. Infect. Dis..

[63] Chertow D.S. (2019). Understanding long-term effects of Ebola virus disease. Nat. Med..

[64] Oleribe O.O., Salako B.L., Ka M.M., Akpalu A., McConnochie M., Foster M., Taylor-Robinson S.D. (2015). Ebola virus disease epidemic in West Africa: Lessons learned and issues arising from West African countries. Clin. Med..

[65] Poni M. (2022). Gov’t Engages Traditional Leaders to End Child Marriage. Number One Citizen Daily Newspaper.

[66] Hanson-DeFusco J., Smith E.G., McMaster A. (2023). Efficacy of civil society organizations to mitigate gender-based sexual violence in schools, in Liberia. J. Civ. Soc..

[67] Hanson J., Faley P., Quinn M. (2017). Analysis of the Liberian Ebola Survivors Support System (ESSS). Integr. J. Glob. Health.

[68] WHO (2015). WHO Meeting on Survivors of Ebola Virus Disease: Clinical Care of Survivors. https://iris.who.int/bitstream/handle/10665/204126/9789241509794_eng.pdf;jsessionid=9C85E2732F8FE0D9740D7E8D1CCB4FD7?sequence=1.

[69] Hanson-DeFusco J., Davis D., Bommareddy M., Olaniyan Z. (2024). Broken Promises: Prolonged Diminished Quality-of-Life among Liberian Ebola Survivors Half a Decade after the 2014-16 West African Outbreak. J. Soc. Behav. Health Sci..

[70] Institutional Review Board (2022). Informed Consent Guidance. https://www.hopkinsmedicine.org/institutional-review-board/guidelines-policies/guidelines/informed-consent-i.

[71] Morreim E.H. (2004). Litigation in Clinical Research: Malpractice Doctrines versus Research Realities. J. Law Med. Ethics.

[72] World Medical Association (WMA) (2013). Declaration of Helsinki as a Statement of Ethical Principles for Medical Research Involving Human Subjects. https://www.wma.net/policies-post/wma-declaration-of-helsinki-ethical-principles-for-medical-research-involving-human-subjects/.

[73] Fallah M. (2015). Clinical Experiences and Data from EVD Survivor Cohorts. https://www.jstor.org/stable/pdf/resrep48066.13.pdf.

[74] Eghrari A.O., Bishop R.J., Ross R.D., Davis B., Larbelee J., Amegashie F., Dolo R.F., Prakalapakorn S.G., Gaisie C., Gargu C. (2021). Characterization of Ebola Virus–Associated Eye Disease. JAMA Netw. Open.

[75] OECD Development Centre (2023). Liberia-Social Institutions and Gender Index. https://www.genderindex.org/.

[76] NIAID (2022). Partnership for Research on Ebola Vaccines in Liberia (PREVAIL) ClinicalTrials.Gov ID NCT02344407. NCT02344407.

[77] NIAID (2022). The Partnership for Research on Ebola Virus in Liberia (PREVAIL). https://www.niaid.nih.gov/diseases-conditions/researching-ebola-africa.

[78] Billioux (2021). Natural History, Disease Progression, and Long-Term Neurologic Sequelae of Ebola Virus Disease Survivors in PREVAIL III. https://www.medifind.com/conditions/seizures/4784/clinical-trial/345631928.

[79] Dwivedi A.K., Mallawaarachchi I., Alvarado L.A. (2017). Analysis of small sample size studies using nonparametric bootstrap test with pooled resampling method. Stat. Med..

[80] Munoz R.T., Pearson L.C., Hellman C.M., McIntosh H.C., Khojasteh J., Fox M.D. (2018). Adverse childhood experiences and posttraumatic stress as an antecedent of anxiety and lower hope. Traumatology.

[81] Cheng Z., Cai M., Tao H., He Z., Lin X., Lin H., Zuo Y. (2016). Efficiency and productivity measurement of rural township hospitals in China: A bootstrapping data envelopment analysis. BMJ Open.

[82] Hall D.E., Hanusa B.H., Stone R.A., Ling B.S., Arnold R.M. (2015). Time Required for Institutional Review Board Review at One Veterans Affairs Medical Center. JAMA Surg..

[83] Keusch M., Mancher B., Keusch G., McAdam K., Cuff P., Mancher M., Busta E.R. (2017). Integrating Clinical Research into Epidemic Response.

[84] Matangwa D. (2022). Who knows the South best but us?. Listening to the Voiceless and Hard to Reach: Creating Space and Opportunities for People-Centered Policy Engagement in East Africa.” UnwokeID: Unethical Issues in 21st Century International Development.

[85] Prevail II Writing Group (2016). A Randomized, Controlled Trial of ZMapp for Ebola Virus Infection. N. Engl. J. Med..

[86] Cook T.D., Shadish W.R., Wong V.C. (2008). Three conditions under which experiments and observational studies produce comparable causal estimates: New findings from within-study comparisons. J. Policy Anal. Manag..

[87] Shadish W.R. (2010). Campbell and Rubin: A primer and comparison of their approaches to causal inference in field settings. Psychol. Methods.

[88] Bolay F.K., Grandits G., Lane H.C., Kennedy S.B., Johnson M.P., Fallah M.P., Wilson B., Njoh W.S., McNay L.A., Hensley L.E. (2019). PREVAIL I Cluster Vaccination Study with rVSVΔG-ZEBOV-GP as Part of a Public Health Response in Liberia. J. Infect. Dis..

[89] Kelly J.D., Weiser S.D., Wilson B., Cooper J.B., Glayweon M., Sneller M.C., Drew C., Steward W.T., Reilly C., Johnson K. (2019). Ebola virus disease-related stigma among survivors declined in Liberia over an 18-month, post-outbreak period: An observational cohort study. PLoS Neglected Trop. Dis..

[90] Bowen L., Smith B., Steinbach S., Billioux B., Summers A., Azodi S., Ohayon J., Schindler M., Nath A. (2016). Survivors of Ebola Virus Disease Have Persistent Neurological Deficits (S53.003). Neurological.

[91] Shantha J.G., Crozier I., Yeh S. (2017). An update on ocular complications of Ebola virus disease. Curr. Opin. Ophthalmol..

[92] Moses S.J., Wachekwa I., Van Ryn C., Grandits G., Pau A., Badio M., Kennedy S.B., Sneller M.C., Higgs E.S., Lane H.C. (2021). The impact of the 2014 Ebola epidemic on HIV disease burden and outcomes in Liberia West Africa. PLoS ONE.

